# A PCOS Paradox: Does Inositol Therapy Find a Rationale in All the Different Phenotypes?

**DOI:** 10.3390/ijms24076213

**Published:** 2023-03-25

**Authors:** Vittorio Unfer, Simona Dinicola, Michele Russo

**Affiliations:** 1 The Experts Group on Inositol in Basic and Clinical Research (EGOI), 00161 Rome, Italy; 2 UniCamillus-Saint Camillus International University of Health Sciences, 00156 Rome, Italy; 3 R&D Department, Lo.Li. Pharma, 00156 Rome, Italy

**Keywords:** PCOS, phenotype D, myo-inositol, endocrine syndrome, dysmetabolism

## Abstract

A recent evaluation of the published data regarding the PCOS topic has highlighted a paradox in the definition of this condition. Even though the name of the syndrome refers to ovarian dysfunction, it seems that patients diagnosed with PCOS are more likely affected by an endocrine and metabolic issue. The term PCOS might not be appropriate to indicate the phenotypes described by the Rotterdam criteria, since the only phenotype with a gynecological issue alone is PCOS phenotype D. This novel perspective regarding how PCOS is currently defined leads the way to a reinterpretation of the entire pathological context and the treatment prescribed, such as inositols. A new point of view on the etiopathogenesis of the disease completely changes the current meaning of PCOS and consequently the therapeutic rationale evaluated to date.

## 1. Introduction

Polycystic ovary syndrome (PCOS) is a widespread reproductive, metabolic, and psychological condition that impacts 5 to 18% of women across their lifespans [1]. Even though the disease was first defined by Stein and Leventhal in 1935 [2], to date, several aspects of this syndrome regarding its etiopathogenesis, diagnosis, and therapies are still debated.

A diagnosis of PCOS is made according to the Rotterdam criteria encompassing all possible combinations of the following features: clinical or biochemical evidence of hyperandrogenism, evidence of oligo-anovulation, and ultrasonographic identification of polycystic ovary morphology (PCOM) [3]. The application of the Rotterdam criteria generates four possible different PCOS phenotypes: phenotype A (classic PCOS), exhibiting evidence of hyperandrogenism, oligo-anovulation, and PCOM; phenotype B (hyperandrogenic anovulation), exhibiting evidence of hyperandrogenism and oligo-anovulation; phenotype C (ovulatory PCOS), exhibiting evidence of hyperandrogenism and PCOM; and phenotype D (non-hyperandrogenic PCOS), exhibiting evidence of oligo-anovulation and PCOM [4].

It seems clear that a condition of hyperandrogenism is frequently observed in women with PCOS and is a common feature of three out of four possible PCOS phenotypes [5]. Hyperandrogenism occurs with clinical evidence and phenotypical manifestations, including hirsutism, acne, alopecia, and seborrhea, or can be biochemically detected with the analysis of circulating androgen levels [6]. Androgen excess correlates with alterations in the menstrual cycle and ovarian physiology and reduces the possibility of pregnancy [7]. An ongoing debate has been drawing scientific attention in the last few years to the need for investigating the risk factors and the potential causes of hyperandrogenic profiles observed in women with PCOS.

Considering that the majority of women with PCOS exhibit insulin resistance (IR) [8,9] or other metabolic issues, including hyperinsulinemia, obesity, and diabetes, it seems reasonable to hypothesize that hyperandrogenism and its dermatological manifestations might derive from metabolic and hormonal imbalances. As such, women affected by PCOS quite often exhibit hyperandrogenism manifestations such as acne, hirsutism, and alopecia, together with an altered menstrual cycle, as markers of an endocrine and metabolic issue rather than a gynecological condition. Interestingly, even those women with PCOS examined by Stein and Leventhal in 1935 were characterized by an enlarged ovarian volume, amenorrhea, and obesity, suggesting the presence of dysmetabolic features [2].

This new perspective supporting the central role of a metabolic imbalance in the ovary in the PCOS scenario could radically change the way the disease is currently understood.

## 2. Redefining PCOS: The Metabolic Alterations

The hypothesis of “metabolic hyperandrogenism” leads to a paradox regarding the term PCOS and its meaning. Indeed, the definition of a woman presenting with a polycystic ovary implies that she has a gynecological disorder. Specifically, the disorder affects the reproductive system, leading to an alteration in ovarian function and the formation of typical ovarian cysts. On the other hand, the term “syndrome” in the medical field indicates a group of symptoms representing the expression of a disease without indicating possible causes or the mechanism of onset [10]. All this considered, it seems unreasonable to use the term PCOS for those phenotypes that originate from a metabolic disorder. At the same time, the term PCOS might not be accurate to indicate a pathology originating from ovarian dysfunction, as it lacks the characteristics of a syndrome. So, what is the real meaning of PCOS? Are the Rotterdam criteria an appropriate tool to make a diagnosis of a polycystic ovary?

It seems that the real meaning of PCOS mainly refers to an endocrine–metabolic disorder, and the widespread presence of the IR phenomenon in these patients further confirms the pivotal involvement of insulin signaling in this syndrome.

On these premises, several authors and endocrine or gynecological societies support the use of different criteria for PCOS diagnosis by questioning the suitability of the application of the Rotterdam criteria. For example, the Androgen Excess and Polycystic Ovary Syndrome Society (AE-PCOS) suggests using otherwise unexplained hyperandrogenism with either oligo-anovulation or PCOM as a criterion [11]. Accordingly, a diagnosis of PCOS is possible in women with hyperandrogenism but without PCOM. Alternatively, the NIH sponsored an evidence-based methodology workshop on PCOS to review the current diagnostic criteria, pointing out the relative importance of PCOM, which is “neither necessary nor sufficient for the diagnosis of PCOS” [12].

These indications further highlight that PCOS, in its current meaning, entails an endocrine–metabolic disorder regardless of ovarian dysfunction. A compelling aspect in this regard is represented by the exclusion of IR from the criteria adopted in the diagnosis of PCOS. Even though about 75% of women with PCOS exhibit IR [13], it is not considered a clear symptom or a diagnostic criterion.

Conditions such as hyperinsulinemia or IR in the context of the female reproductive apparatus allow the introduction of another concept, appropriately indicated as the ovarian paradox. Indeed, as the ovaries remain sensitive to insulin signaling, hyperinsulinemia due to systemic IR may contribute to increased androgen secretion [14] and lead to ovulatory dysfunction [15].

Considering the primary importance of metabolic alterations in this syndrome, it might be questioned whether the phenotypes based on the Rotterdam criteria are appropriate to describe a condition originating from ovarian dysfunction. Hence, women with PCOS phenotype B (hyperandrogenic anovulation) lack evidence of PCOM and do not exhibit a clear gynecological issue, while the presence of hyperandrogenism in phenotypes A (classic PCOS) and C (ovulatory PCOS) suggests a plausibly imbalanced metabolism that may or may not co-exist with an ovary issue.

**Figure 1:**
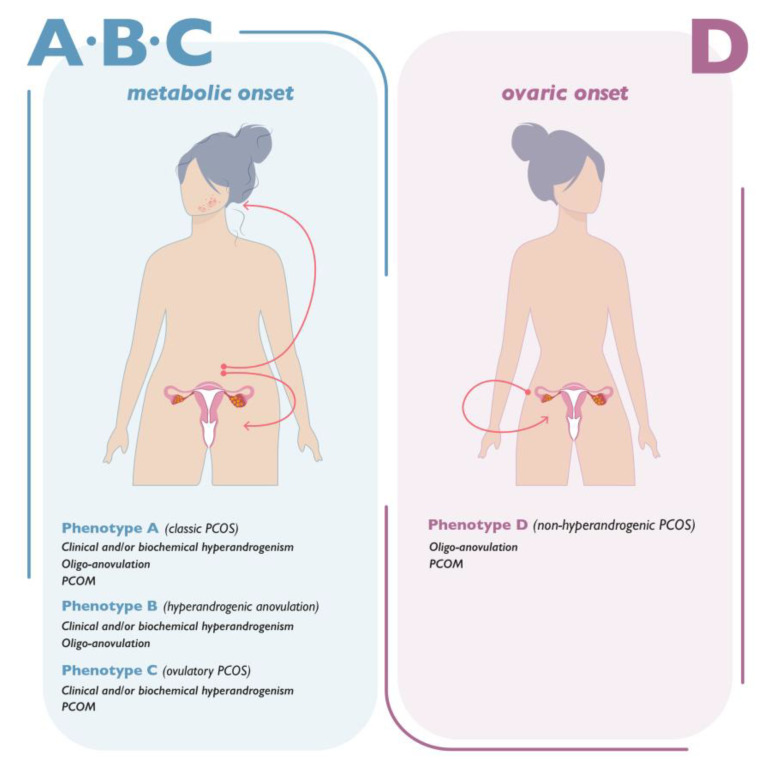
PCOS phenotypes. PCOS (polycystic ovary syndrome) and PCOM (polycystic ovarian morphology). Schematic illustration of the onset of the PCOS condition in the different phenotypes. Phenotypes A, B, and C share the hyperandrogenism condition and a metabolic onset of the syndrome, which leads to the occurrence of the esthetic manifestations of hyperandrogenism (acne, alopecia, hirsutism, and seborrhea), oligo-amenorrhea, and ovarian cysts. Phenotype D exhibits an ovarian onset of the condition, which directly causes an alteration in the menstrual cycle and the formation of ovarian cysts.

In light of this consideration, it seems reasonable that phenotype D identifies the only gynecological condition and an actual ovarian syndrome (Figure 1).

## 3. Alternative Classification for PCOS Phenotypes

As previously discussed, factors such as insulin signaling and IR are involved in androgen excess and in the development of hyperandrogenism. In a study by Moghetti and colleagues [16], different phenotypes of PCOS were evaluated in relation to IR by using a glucose clamp to measure the secretion of insulin and the resistance phenomenon in the body. The authors discovered that sensitivity to insulin was reduced in PCOS phenotypes A and C, but not in phenotype D or healthy women. Lower sensitivity to insulin translates into higher IR, so women with phenotype D PCOS exhibit a normal metabolic profile, unlike phenotypes A and C.

The findings of this study demonstrate the differences in the metabolic background of the various PCOS phenotypes. The characteristics of non-hyperandrogenic phenotype D PCOS appear in a separate group within the syndrome and include a metabolic profile very similar to that of healthy women. Indeed, the syndrome in these patients seems not to derive from or be associated with metabolic deregulation [16]. As phenotype D PCOS does not share a pathological profile with the other phenotypes, one should hypothesize a different etiology of the pathology for these women.

Recently, an in vitro study revealed that the stimulation of 3D follicles from mouse models with insulin-like growth factor-1 (IGF-1) causes the arrest of follicle growth when excessive IGF-1 amounts were used [17]. The authors investigated the effect of three different concentrations of IGF-1 and observed that the treatment with the highest concentration inhibited follicle growth. The results of this study surprisingly describe a novel pathway that might lead to the premature arrest of follicle growth and consequently to the formation of cysts in the ovary. As the signaling of IGF-1 and the balance of androgens are unrelated, IGF-1 upregulation could be involved in the etiopathogenesis in women with phenotype D PCOS. Since women with phenotype D PCOS are non-hyperandrogenic, they exhibit a pathology separate from metabolic alterations, suggesting that the development of ovarian cysts in non-hyperandrogenic PCOS follows a different mechanism from that in hyperandrogenic PCOS.

Given that women with phenotype D PCOS seem to be the only group exhibiting a disease of gynecological origin, it might be questioned whether the definition of PCOS really applies to the other phenotypes with a metabolic syndrome. If not, what should we consider as an alternative classification?

Hyperandrogenic PCOS is frequently associated with hirsutism, acne, alopecia, or excessive weight gain, but these symptoms seem more likely to be epiphenomena of a metabolic alteration, which leads to gynecological issues. These canonical features do not represent the origin of the problem of PCOS, since they refer to the manifestation of the pathology rather than identify a polycystic morphology of the ovary. If we indicate the condition of the polycystic ovary, it might be argued that PCOS is not a comprehensive definition, particularly as it is understood in the scientific community.

Supporting evidence for this view can be found in a recent definition of “male PCOS” published by several authors [18]. The term PCOS in this case obviously refers to metabolic disorders with phenotypic signs that include alopecia, elevated BMI, erectile dysfunction, fertility problems, and prostate hyperplasia [19]. As previously discussed, all the phenotypic features characterizing PCOS in men reflect the existence of an endocrine and metabolic syndrome, which, as a consequence, produces aberrations in the reproductive apparatus.

**Figure 2:**
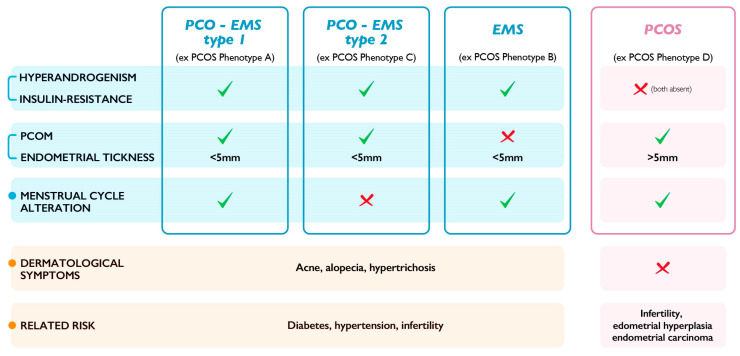
New classification for PCOS. PCOS (polycystic ovary syndrome), EMS (endocrine–metabolic syndrome), PCOM (polycystic ovarian morphology), and PCO (polycystic ovary). New combinations of different parameters indicate the phenotypes of an innovative PCOS classification. PCOS phenotype A is updated as “PCO-EMS type 1” with the presence of both hyperandrogenism and insulin resistance, the presence of PCOM and an endometrial thickness < 5 mm, and the presence of menstrual cycle alterations; PCOS phenotype C is updated as “PCO-EMS type 2” with the presence of both hyperandrogenism and insulin resistance, the presence of an endometrial thickness < 5 mm and PCOM, and the absence of menstrual cycle alterations; PCOS phenotype B is updated as “EMS” with the presence of both hyperandrogenism and insulin resistance, the presence of an endometrial thickness < 5 mm and the absence of PCOM, and the presence of menstrual cycle alterations. Subjects presenting PCO-EMS type 1, PCO-EMS type 2, and EMS share the same dermatological symptoms (acne, alopecia, and hypertrichosis) and the same related risk (diabetes, hypertension, and infertility). PCOS phenotype D is updated as “PCOS” with the absence of both hyperandrogenism and insulin resistance, the presence of PCOM and an endometrial thickness > 5 mm, and the presence of menstrual cycle alterations. Subjects with PCOS do not exhibit dermatological symptoms and share related risks (infertility, endometrial hyperplasia, and endometrial carcinoma) differing from those in subjects with EMS. ✓: feature present; X: feature absent.

On these premises, the definition of “PCOS” should be appropriate only to indicate women with phenotype D, while the remaining phenotypes should be indicated as different types of an “endocrine-metabolic syndrome”. An innovative classification might also be proposed, defined by different criteria and parameters that could describe the PCOS phenotypes as defined today, with new feature combinations considered for the diagnosis and a new nomenclature for these phenotypes (Figure 2).

This aspect completely changes the perception of the etiopathogenesis of the PCOS condition as it is defined today and implies the need to revise the diagnostic assessment and the therapeutic approach. Indeed, if this new vision of an endocrine–metabolic syndrome applies, it could also be postulated that the term PCOS does not properly indicate a gynecological pathology and a gynecologist should not be the referred specialist. As alterations in the ovary region are not involved in the generation of the phenotypes described by the Rotterdam criteria, except for phenotype D, the presence of an endocrine–metabolic imbalance suggests that a diagnosis of PCOS in its current meaning should be made by an endocrinologist or, alternatively, by a gynecologist specializing in endocrinological gynecology.

## 4. Changing the Therapeutic Rationale

To date, the therapeutic approaches commonly recommended for PCOS patients are (1) lifestyle changes to increase physical activity and correct caloric intake; (2) the use of oral combined pills (OCPs) to reverse the hormonal imbalance; (3) the use of insulin sensitizers, such as metformin or inositols, to improve the metabolic profile [20,21].

However, in this novel view of phenotypes A, B, and C as endocrine–metabolic syndromes, the current therapeutic rationale should be revised, as a misunderstanding of the disease etiology leads to incorrect therapeutic indications. If we are dealing with metabolic deregulation, there is no need to intervene by targeting the ovary. This new point of view regarding PCOS means that evaluating therapeutic approaches that may act at the metabolic level is mandatory.

Frequently, the first-line medical treatment for women with PCOS is OCPs [22]. They are recommended to reduce the hyperandrogenic signs of the syndrome and to regulate the menstrual cycle [23], and OCPs are widely prescribed for the teenage population, where the dermatological impact of the syndrome is particularly burdensome [20]. However, OCPs only treat the hyperandrogenism symptoms of the syndrome, without addressing ovarian issues or metabolic alterations. Moreover, hyperandrogenism manifestations are not observed in women with phenotype D PCOS, and these OCPs might be not considered a universal approach.

It is also important to recall that there are also many concerns regarding the safety of the long-term use of these pills [24], and evaluating alternative treatments might be appropriate.

In this context, the first remedy to be evaluated and recommended should always be a lifestyle intervention [25]. Usually, a change in dietary habits has a beneficial effect on subjects with PCOS, who often exhibit improvements in their lipid profiles, systemic inflammatory state, insulin signaling, and sometimes ovulatory issues [26,27]. Furthermore, an increase in physical activity has also been evaluated, with the results highlighting that the improvement of the diet in association with physical exercise is effective in the management of PCOS [28,29]. By acting on the metabolic profile, diet and physical activity can improve the condition of women with PCOS affected by dysmetabolism.

Since the described metabolic alterations, particularly IR, impact a large portion of PCOS subjects, insulin-sensitizing agents are a therapeutic option [30]. For example, metformin improves IR in subjects with PCOS or type-2 diabetes mellitus and reduces androgen levels and related esthetic issues such as acne and increased BMI. Furthermore, it has beneficial effects on menstrual cycle regularization [31,32,33]. However, a metformin-based clinical approach is not free of concerns, including its lack of efficacy or specificity in some patients, side effects observed in long-term treatments, and the safety of its administration during pregnancy [34,35].

Alternative insulin sensitizers have been evaluated, and several authors have described significant improvements in PCOS parameters with inositol administration [36].

Two stereoisomers, myo-inositol (myo-Ins) and D-chiro-inositol (D-chiro-Ins), are naturally present in the ovaries and in the follicular fluid and participate in follicular development by mediating hormonal activities, including insulin signaling. Despite the chemical similarities and their synergistic effect on insulin sensitivity, they exert different effects on the ovary [37].

The rationale supporting the importance of inositols in the PCOS scenario relies on the demonstration that specific myo-Ins/D-chiro-Ins ratios maintain reproductive functions, while alterations lead to an aberrant ovulation process and to androgen overproduction. Oral supplementation with inositols, indeed, represents a valid approach to counteracting the signs of PCOS by improving metabolic alterations in patients and reducing the manifestations of hyperandrogenism [38,39].

In particular, in overweight–obese women, a formulation with both stereoisomers, based on the physiological ratio of myo-Ins/D-chiro-Ins (40:1) observed in the plasma, can restore the cellular content of myo-Ins in the ovary while maintaining the correct ratio with D-chiro-Ins [40]. The treatment revealed an improvement in IR and in metabolic and endocrine parameters [41,42].

Interestingly, D-chiro-Ins elicits positive activity in the ovary when used at the right concentrations for a brief treatment period by regulating steroidogenic enzyme genes in human granulosa cells, lowering the expression of both aromatase and cytochrome P450, and increasing testosterone levels in theca cells [43,44]. Conversely, it can aggravate the PCOS condition if administered in excessive amounts or for prolonged periods [45,46].

Myo-Ins, in particular, plays a pivotal role in sustaining the entire reproduction process [47] and improving gestation outcomes in PCOS patients [48]. Indeed, myo-Ins administration in women with PCOS enhances the oocyte quality and competence [49] and ameliorates the endometrial status, thereby improving the fertilization phase [50] and thus resulting in an overall reduction in possible pregnancy complications [51]. 

Recent data also demonstrate that the combination of inositols, especially myo-Ins, with probiotic molecules such as α-lactalbumin enhances intestinal adsorption, improves the gastric environment, reduces the inflammatory state, and enhances the beneficial activity of myo-Ins in the PCOS condition [38,52].

The efficacy of inositol supplementation in PCOS scenarios has been endorsed worldwide for more than 20 years [53], but interestingly, therapy with inositols is not appropriate for patients exhibiting phenotype D PCOS, since they are not affected by hyperandrogenism and do not always experience dysmetabolism [16].

This aspect regarding a sub-group of PCOS patients raises questions concerning the best clinical approach to be considered for the treatment of this condition [54]. Currently, the four PCOS phenotypes are not clinically or biochemically distinct concerning the treatment recommendation, and they could also change over the patient’s lifetime. Hence, a patient-tailored treatment should be considered to address the clinical features of the different phenotypes of PCOS that are defined today [55].

## 5. Final Considerations on Future Directions

Moreover, the hypothesis of a redefinition of PCOS also suggests the need for a reinterpretation of the literature evidence published. Among a huge number of scientific articles on PCOS, reports on the differentiation of the subject phenotypes are very rare. Patients enrolled in clinical studies, for instance, are not divided into sub-groups according to their phenotypes or other PCOS features. As a result, the various phenotypes are mixed together in the study groups: hyperandrogenic patients are included together with non-androgenic patients, or ovulatory and anovulatory subjects are included in the same group. The heterogeneity of study groups might alter the results of those studies and might change the effectiveness of the treatment investigated.

In light of these considerations, the existence of a paradox regarding the definition of PCOS, where a gynecologic etiology could be ruled out, appears evident, and the blurred edges of the pathology and therapy have yet to be properly defined.

## Data Availability

Not applicable.
